# CD5 Surface Expression Marks Intravascular Human Innate Lymphoid Cells That Have a Distinct Ontogeny and Migrate to the Lung

**DOI:** 10.3389/fimmu.2021.752104

**Published:** 2021-11-18

**Authors:** Arlisa Alisjahbana, Yu Gao, Natalie Sleiers, Elza Evren, Demi Brownlie, Andreas von Kries, Carl Jorns, Nicole Marquardt, Jakob Michaëlsson, Tim Willinger

**Affiliations:** ^1^ Center for Infectious Medicine, Department of Medicine Huddinge, Karolinska Institutet, Karolinska University Hospital, Stockholm, Sweden; ^2^ Department of Clinical Science, Intervention and Technology, Karolinska Institutet, Stockholm, Sweden; ^3^ Department of Transplantation Surgery, Karolinska University Hospital, Stockholm, Sweden

**Keywords:** innate lymphoid cells (ILC), lung, migration, ontogeny, humanized mice

## Abstract

Innate lymphoid cells (ILCs) contribute to immune defense, yet it is poorly understood how ILCs develop and are strategically positioned in the lung. This applies especially to human ILCs due to the difficulty of studying them *in vivo*. Here we investigated the ontogeny and migration of human ILCs *in vivo* with a humanized mouse model (“MISTRG”) expressing human cytokines. In addition to known tissue-resident ILC subsets, we discovered CD5-expressing ILCs that predominantly resided within the lung vasculature and in the circulation. CD5^+^ ILCs contained IFNγ-producing mature ILC1s as well as immature ILCs that produced ILC effector cytokines under polarizing conditions *in vitro*. CD5^+^ ILCs had a distinct ontogeny compared to conventional CD5^-^ ILCs because they first appeared in the thymus, spleen and liver rather than in the bone marrow after transplantation of MISTRG mice with human CD34^+^ hematopoietic stem and progenitor cells. Due to their strategic location, human CD5^+^ ILCs could serve as blood-borne sentinels, ready to be recruited into the lung to respond to environmental challenges. This work emphasizes the uniqueness of human CD5^+^ ILCs in terms of their anatomical localization and developmental origin compared to well-studied CD5^-^ ILCs.

## Introduction

Innate lymphoid cells (ILCs) are an emerging family of immune cells with diverse roles in tissue homeostasis, barrier maintenance, host-microbiota crosstalk, and immunity to pathogens (1–5). As innate counterparts of CD4^+^ T helper cells, ILCs are divided into three subsets based on signature transcription factors and effector cytokines: (1) ILC1s express T-BET and produce interferon gamma (IFNγ); (2) ILC2s express GATA-3 and produce interleukin-5 (IL-5) and IL-13; (3) ILC3s express RORγt and produce IL-17 and/or IL-22. ILCs require IL-7 for their development and thus are characterized by the expression of IL-7Rα (CD127), while lacking lineage (Lin) markers that define other cell lineages, such as related cytotoxic natural killer (NK) cells (6–8).

One aspect of ILC biology that is difficult to study in human *ex-vivo* samples, is the migration of ILCs despite the importance of ILC migration in tissue homeostasis and inflammation (9, 10). Mouse studies have shown that ILCs are able to move within tissues and to traffic to organs *via* the blood during development and inflammation (11–16). In contrast, the migration of human ILCs and their tissue niches remain much less explored. To overcome this limitation, we are using humanized mouse models, which allow studies of the human immune system in an *in-vivo* setting (17–19). In particular, we previously developed a humanized mouse model, named “MISTRG”, which expresses the human cytokines and proteins M-CSF, IL-3/GM-CSF, SIRPα, and TPO through gene knock-in (20–22). After transplantation with human hematopoietic stem and progenitor cells (HSPCs), MISTRG mice support the reconstitution of a human immune system in the mouse host, including the development of human NK cells (21). Human ILCs other than NK cells are also present in MISTRG mice and therefore this model offers the opportunity to study the biology of human ILCs *in vivo*.

In mice, ILCs are considered to be mostly tissue-resident (23), whereas circulating ILC subsets, mainly ILC2s and CD117^+^ ILC precursors (ILCPs), have been shown to be present in humans (24–26). This led to the concept of local ILC-poiesis, where circulating ILCPs migrate into tissues to give rise to mature ILC subsets (27). However, it is unclear whether additional human ILCPs exist in the circulation.

CD5 is a cell surface protein mainly expressed by T lymphocytes and has an inhibitory function in antigen receptor signaling (28, 29). Accordingly, CD5 has been used in several studies to exclude T cells when analyzing ILCs (24, 26, 30–32). However, it has been discovered that ILC1s express T cell-associated molecules, including CD5 (33–38). Subsequently, CD5^+^ ILCs were found to be present in human thymus and umbilical cord blood (34). Furthermore, the Spits’ laboratory demonstrated that immature CD5^+^ ILC2s were able to differentiate into mature cytokine-producing ILC2s *in vitro* (34). More recently, ILC1-like cells were identified in human cord blood that expressed CD5 and contained precursors for mature KIR^+^NKG2A^-^ NK cells (39). These studies indicate that human CD5^+^ ILCs are heterogeneous and may be functionally distinct from conventional CD5^-^ ILCs that have been extensively studied. However, the development and migration of human CD5^+^ ILCs remains poorly understood.

Here we investigated human CD5^+^ ILCs in humanized mice *in vivo* with a focus on the lung. We demonstrate that CD5^+^ ILCs derived from CD34^+^ HSPCs are distinct from well-studied CD5^-^ ILCs in terms of their developmental origin, migration, and tissue localization. Specifically, we identified CD5-expressing human ILCs in the lung that had a predominantly intravascular localization. CD5^+^ ILCs followed a different developmental path than conventional ILCs as they occupied a distinct anatomical niche and likely originated from the thymus, liver and/or spleen. Finally, circulating CD5^+^ ILCs were composed of not only mature ILC1s, but also actively dividing immature ILCs that produced different ILC effector cytokines *in vitro*. Due to their intravascular localization, human CD5^+^ILCs could function as blood-borne sentinels that are poised to respond to local and systemic environmental challenges.

## Materials and Methods

### Mice

MISTRG mice homozygous for the human genes encoding M-CSF, IL-3/GM-CSF, SIRPα, and TPO in the *Rag2* ^-/-^ *Il2rg* ^-/-^ background were previously described (21). MISTRG mice were used under Material Transfer Agreements with Regeneron Pharmaceuticals and Yale University. For this study, we used an improved version of MISTRG mice with a *SIRPA* knock-in allele (40), instead of *SIRPA* transgene as in the original MISTRG mice. For transplantation with human CD34^+^ cells, MISTRG mice heterozygous for *SIRPA* knock-in were used (both males and females). Heterozygous mice were derived from breeding MISTRG mice (homozygous for *SIRPA*) with MITRG mice (lacking the *SIRPA* knock-in allele) (21). MISTRG mice were re-derived by embryo transfer at Karolinska Institutet and maintained in individually ventilated cages under specific pathogen-free conditions without any prophylactic antibiotics. All mouse experiments were performed in accordance with protocols approved by the Linköping Animal Experimentation Ethics Committee (#29-15, 03127-2020).

### Human Tissues

Umbilical cord blood and buffy coats were obtained from Caesarean sections and the Blood Bank at Karolinska University Hospital Huddinge, respectively. Human lung and spleen tissue was obtained from deceased organ donors through the Transplantation Clinic at Karolinska University Hospital Huddinge, where the lungs were not used for lung transplantation. Donors included 3 males and 2 females who were 25-83 years old. Causes of death were cardiac arrest, hypoxic brain damage, intracranial hemorrhage as well as subdural and subarachnoid hematoma. The collection of all human tissues was approved by local Ethical Review Boards at Karolinska Institutet (#2006/229-31/3, 2015/1368-31/4, 2015/2122-32, 2016/1415-32, 2019-05016). Informed consent was obtained from all tissue donors following verbal and written information and the investigations were conducted according to the Declaration of Helsinki.

### Cell Isolation From Human Blood and Tissues

Peripheral blood mononuclear cells were isolated from cord blood and buffy coats using density gradient centrifugation with Lymphoprep (Fisher Scientific). For isolation of mononuclear cells from human lungs, the lung tissue was cut into small pieces and digested for 30 minutes at 37°C in RPMI 1640 (supplemented with 100 U/ml Penicillin, 50 μg/ml streptomycin, 1 mM L-glutamine) containing 0.25 mg/mL collagenase II (Sigma), and 0.2 mg/mL of DNAse I (Roche). Digested tissue was washed with RPMI 1640 supplemented with 10% fetal calf serum (FCS) as well as 100 U/ml Penicillin, 50 ug/ml streptomycin, and 1 mM L-glutamine. Cells were then filtered through 70 μm and 40 μm cell strainers with a syringe plunger. Finally, after washing, mononuclear cells were obtained by density gradient centrifugation with Lymphoprep. For isolation of mononuclear cells from human spleens, a 2-3 cm piece of spleen was mechanically mashed and passed through a cell strainer.

### Transplantation With Human CD34^+^ Cells

For transplantation with human HSPCs, CD34^+^ cells were isolated from pooled cord blood by density gradient centrifugation and positive immunomagnetic selection using a CD34^+^ microbead kit (Miltenyi Biotec). Newborn MISTRG mice (3-5 days old) were transplanted with 1 x 10^5^ human CD34^+^ cells (usually >90% purity) by intrahepatic injection as previously described (22). HSPCs were pooled from several donors for transplantation. Mice did not receive any irradiation as pre-conditioning before transplantation. At ~7 weeks post-transplantation, blood was collected to determine engraftment of MISTRG mice with human CD45^+^ hematopoietic cells by flow cytometry. Mice were generally used for experiments at 7-12 weeks after transplantation with human CD34^+^ cells, except for the kinetics experiments where mice were analyzed also at 3-5 weeks post-transplantation.

### Isolation of Immune Cells From MISTRG Mice

Lungs were perfused with cold PBS, minced into small pieces, and digested for 60 minutes at 37°C in digestion media composed of RPMI 1640 supplemented with 5% FCS, 0.2 mg/mL collagenase IV (Sigma), and 0.02 mg/mL of DNAse I (Sigma). After digestion, cells were mechanically dissociated by sequentially passing them through 18G and 20G needles attached to a syringe. Cells were then filtered and subjected to density gradient centrifugation with Lymphoprep (Fisher Scientific). Bronchoalveolar lavage (BAL) fluid was collected by inflating the lungs three times with 0.8 mL PBS *via* a catheter inserted into the trachea. BAL fluid was then centrifuged, the pellet resuspended in RPMI 1640/5% FCS, and BAL cells purified for flow cytometry by density gradient centrifugation. Livers were excised, mechanically crushed into small pieces, and digested for 60 minutes at 37°C in digestion media composed of RPMI 1640 supplemented with 5% FCS, 0.2 mg/mL collagenase IV (Sigma), and 0.02 mg/mL of DNAse I (Sigma). After washing with RPMI 1640/5% FCS and low-speed centrifugation (300 rpm for 3 minutes at 4°C) to remove hepatocytes, the supernatant was saved. Cells were then pelleted by centrifugation (1,700 rpm for 10 minutes at 4°C) and subjected to density gradient centrifugation with 27.5% Optiprep (Abbott Rapid Diagnostics) and red blood cell lysis. Blood was taken by cardiac puncture and diluted in 200 units/mL heparin (Sigma). Erythrocytes were removed using red blood cell lysis buffer (obtained from Karolinska University Hospital) and the remaining immune cells stained for flow cytometry analysis. Spleens and thymi from MISTRG mice were mashed using a syringe plunger and passed through a 70 µm filter before red blood cell lysis. To isolate bone marrow cells from MISTRG mice, hind legs were harvested and the bones were cleaned. After cutting the bone ends off, bone marrow cells were flushed out using a syringe, filtered, and treated with red blood cell lysis buffer. Isolated single cells were washed with RPMI 1640, counted, and then either stained directly for flow cytometry or stored overnight in R10 media (RPMI 1640 with 10% FCS and 1% L-glutamine) at 4°C prior to intracellular cytokine staining.

### Intravascular Cell Labelling

HSPC-engrafted MISTRG mice were injected intravenously with 2 μg of PE-conjugated anti-human CD45 antibody (Biolegend, clone HI30) to label human hematopoietic cells in the blood and the lung vasculature. Mice were sacrificed 5 minutes after injection and lungs harvested without prior perfusion. Lung immune cells were isolated as above and stained with APC-Cy7-conjugated anti-human CD45 antibody and other antibodies *ex vivo* for flow cytometry as described below. As a positive and negative control, cells from blood and BAL fluid were used, respectively. Only lung samples from mice with successful intravascular labelling (>90% CD45-PE^+^ in blood) were used for analysis.

### Flow Cytometry

Single-cell suspensions from blood and tissues of MISTRG mice or from human samples were stained with fluorochrome- or biotin-labeled antibodies (see **Supplementary Table 1**) in 100 μl FACS buffer (PBS/2% FCS) for 30 minutes on ice, followed by secondary staining with streptavidin-Brilliant Violet 711 (BD Biosciences) for 20 minutes on ice. After surface staining, cells were stained with fixable viability dye-eFluor506 (eBioscience) according to the manufacturer’s instructions. For detection of intracellular proteins, cells were first stained with surface antibodies and viability dye as above. Then cells were fixed and permeabilized using the Foxp3/Transcription Factor Staining kit (eBioscience) according to manufacturer’s protocol before staining with antibodies against transcription factors or Ki67. For intracellular CD3 and TCRab staining, cells were fixed and permeabilized with the Cytofix/Cytoperm kit (BD Biosciences) according to the manufacturer’s instructions. Matched isotype antibodies were used as controls. For intracellular cytokine staining, cells were plated into 24-well plates at a cell density of 2.5-5 x 10^6^ cells/ml (5-10 x 10^6^ cells per well). Cells were then stimulated for 3 hours at 37°C with 100 ng/ml phorbol 12-myristate 13-acetate (PMA) (Sigma) and 1 μg/ml ionomycin (Sigma) in R10 medium containing a 1:1,000 dilution of Golgi Plug (BD Biosciences). After surface and viability staining, cells were fixed, permeabilized, and intracellular cytokine staining performed using the Cytofix/Cytoperm kit (BD Biosciences) according to the manufacturer’s instructions. Stained cells were acquired on a LSR II Fortessa flow cytometer (BD Biosciences), and data were analyzed with FlowJoV10 software.

### *In-Vitro* Differentiation of Human ILCs

Peripheral blood mononuclear cells from buffy coat were enriched for ILCs using a negative selection protocol for CD3, CD14, CD16, and CD19 using Mojosort Streptavidin Nanobeads (Biolegend) as described by Krabbendam et al. (41). CD5^-^CD7^+^ and CD5^+^CD7^+^ ILCs were then purified from CD127^+^Lin^-^CD3^-^TCRαβ^-^CD94^-^ cells using a MA900 cell sorter (Sony Biotechnology). Each ILC subset (1,000 cells per well) was co-cultured with OP9-DL1 cells (3,000 per well) in a 96-well U-bottom plate. OP9-DL1 cells were maintained in IMDM/10% FCS with penicillin/streptomycin before co-culture with ILCs. Cells were co-cultured for one week in IMDM supplemented with Yssel’s media and 2% human serum, together with 20 ng/ml IL-2 and 20 ng/ml IL-7 (Miltenyi Biotech). For polarizing conditions the following cytokines (Peprotech) were added (all at 20 ng/ml unless stated otherwise) (1) ILC1s: IL-1β and IL-12; (2) ILC2s: IL-4, IL-25, IL-33, and TSLP; (3) ILC3s: IL-1β and IL-23. Cytokines were replenished at day 5 of culture. At day 7 of culture, cells were harvested, stimulated with PMA and ionomycin and stained for intracellular cytokines as described above.

### Quantification and Statistical Analysis

Statistical parameters including number of biological replicates and repeat experiments, data dispersion and precision measures (mean and standard error of the mean (SEM)), and *P* values for statistical significance (*α *= 0.05) are reported in Figures and Figure Legends. Student’s *t* test was used to determine statistical significance between two groups. Multigroup comparisons were performed using one-way ANOVA followed by *post hoc* testing using Tukey’ Multiple Comparison Test. Statistical analysis was performed using GraphPad Prism 8.

## Results

### Human CD5^+^ ILCs Are Present in the Lung and Peripheral Blood

**Figure 1:**
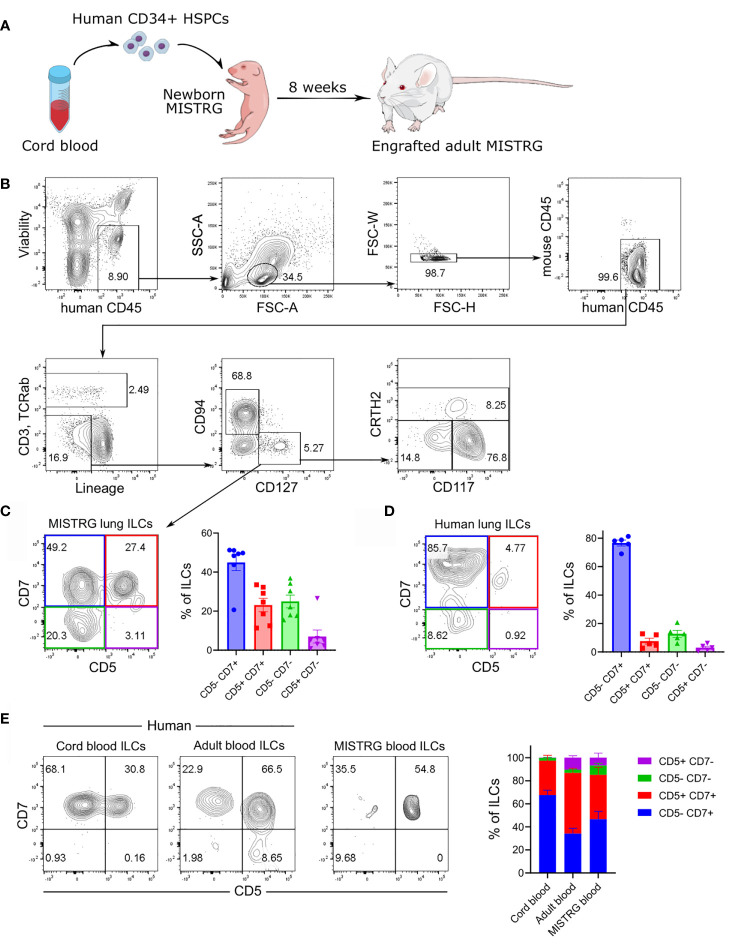
Human CD5^+^ ILCs are present in the lung and peripheral blood. **(A)** Engraftment of newborn MISTRG mice with human CD34^+^ HSPCs from cord blood. **(B)** Gating strategy for the flow cytometric analysis of human ILCs in the lung of HSPC-transplanted MISTRG mice. Total ILCs were gated as viable human CD45^+^CD3^-^TCRαβ^-^CD94^-^CD127^+^ cells that were negative for lineage (Lin) markers (CD11c, CD14, CD19, CD34, CD123, FceRI) and mouse CD45. **(C, D)** CD5 and CD7 surface expression on human lung ILCs. Frequencies of CD5/CD7-expressing ILC subsets in the lung of HSPC-engrafted MISTRG mice (n = 7) and in human lung tissue (n = 5) are shown in **(C, D)**, respectively. **(E)** Frequencies of CD5/CD7-expressing ILC subsets in human cord blood, human adult blood, and in the blood of HSPC-transplanted MISTRG mice was determined by flow cytometry (n = 7-11). Data are represented as mean ± SEM. Data are from at least three independent experiments. **(A)** was adapted from Mind the Graph.

We previously showed that MISTRG mice transplanted with human HSPCs harbor human NK cells in several tissues, including the lung (21). In contrast, human ILC subsets derived from HSPCs have not been characterized in MISTRG mice. To investigate human lung ILCs *in vivo*, we transplanted newborn MISTRG mice with human CD34^+^ cells containing HSPCs (**Figure 1A**), as in our earlier studies (21, 22, 42). Apart from CD127^-^CD94^+^ NK cells, MISTRG mice engrafted with human CD34^+^ HSPCs harbored different types of CD127^+^CD94^-^ ILCs in the lung that did not express TCRαβ and CD3 on the cell surface and lacked the Lin markers CD11c, CD14, CD19, CD34, CD123, and FceRI (**Figure 1B**). These CD127^+^CD94^-^ ILCs consisted of CD117^+^ ILCPs/ILC3s (24, 43–45), as well as CRTH2^+^ ILC2s (46, 47) and CD117^-^CRTH2^-^ ILC1s (48, 49). Furthermore, flow cytometry revealed a population of human CD127^+^CD3^-^TCRαβ^-^CD94^-^ ILCs in the lung of HSPC-engrafted MISTRG mice that expressed both CD5 and CD7 on the cell surface (**Figure 1C**). CD5^+^CD7^+^ ILCs were also present in the human lung, although they were less prevalent than in HSPC-engrafted MISTRG mice (**Figure 1D** and **Supplementary Figure 1A**). In contrast to ILCs, human CD127^-^CD94^+^ NK cells lacked CD5 expression (**Supplementary Figure 1B**). Consistent with previous reports (34, 39), we detected CD5^+^CD7^+^ ILCs also in human cord blood (**Figure 1E**). Human CD5^+^CD7^+^ ILCs were prevalent in adult blood, as reported previously (35, 50), and they were also present with a similar frequency in the blood of HSPC-engrafted MISTRG mice (**Figure 1E**). Similar to earlier studies (34, 35, 39, 50), CD5^+^CD7^+^ ILCs expressed intracellular CD3, but lacked intracellular TCRαβ protein (**Supplementary Figure 2**), confirming that they were distinct from T cells. These data demonstrate that human CD5^+^CD7^+^ ILCs represent a sizeable population in the circulation and the lung, prompting us to further investigate their biology.

### CD5^+^ ILCs in the Lung Contain Both Mature ILC1s and Functionally Immature ILCs

**Figure 2:**
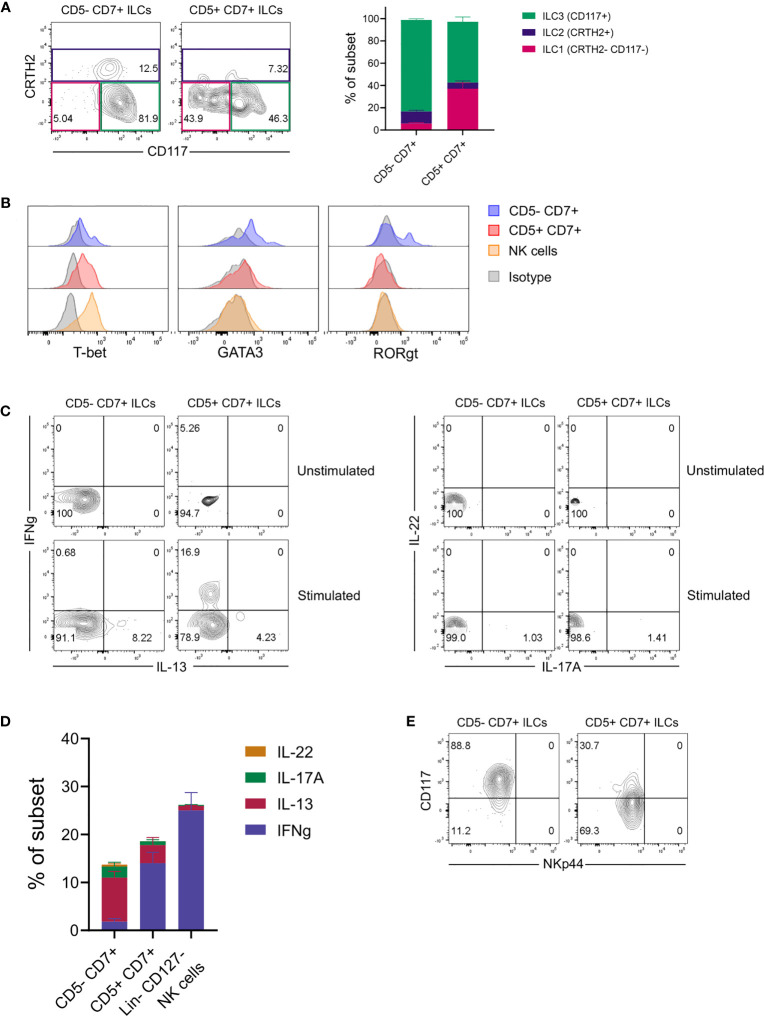
CD5^+^ ILCs in the lung contain both mature ILC1s and functionally immature ILCs. **(A)** Frequencies of ILC1s (CD117^-^CRTH2^-^), ILC2s (CRTH2^+^), and ILC3s (CD117^+^CRTH2^-^) among ILC subsets based on expression of CD5 and CD7 in the lung of HSPC-engrafted MISTRG mice (n = 7). ILCs were gated as human CD45^+^Lin^-^CD3^-^TCRαβ^-^CD94^-^CD127^+^ cells as in **Figure 1B** and then as CD5^-^CD7^+^ or CD5^-^CD7^+^ ILCs as in **Figure 1C**. **(B)** Intracellular expression of signature transcription factors (T-BET, GATA3, RORγt) in human CD5^-^CD7^+^ ILCs, CD5^+^CD7^+^ ILCs, and NK cells isolated from the lung of HSPC-engrafted MISTRG mice. ILCs were gated as human CD45^+^Lin^-^CD3^-^TCRαβ^-^CD127^+^ cells and NK cells were gated as human CD45^+^Lin^-^CD3^-^TCRαβ^-^CD127^-^CD56^+^ cells. Matched isotype antibodies were used as controls. **(C)** Intracellular IFNγ and IL-13 as well as IL-17A and IL-22 production by human CD5^-^CD7^+^ and CD5^-^CD7^+^ lung ILCs from HSPC-engrafted MISTRG mice. Cells were stimulated with PMA/ionomycin or left unstimulated. **(D)** Frequencies of effector cytokine-producing producing cells in each CD5/CD7-expressing ILC subset and NK cells following PMA/ionomycin stimulation (n = 5). NK cells were gated as human CD45^+^Lin^-^CD3^-^TCRαβ^-^CD127^-^ cells. **(E)** NKp44 surface expression on human CD5^-^CD7^+^ ILCs and CD5^+^CD7^+^ ILC in the lung of HSPC-engrafted MISTRG mice. ILCs were gated as human CD45^+^Lin^-^CD3^-^TCRαβ^-^CD94^-^CD127^+^ cells as in **Figure 1B**. Data represent mean ± SEM and are representative of at least two independent experiments.

Further flow cytometric analysis showed that conventional CD5^-^CD7^+^ ILCs in the lungs of engrafted MISTRG mice mainly comprised CD117^+^ ILCPs/ILC3s and CRTH2^+^ ILC2s (**Figure 2A**). In contrast, CD117^-^CRTH2^-^ ILC1s were more abundant among human CD5^+^CD7^+^ ILCs (**Figure 2A**). To gain additional insights into the identity and heterogeneity of human CD5^+^CD7^+^ ILCs, we performed intracellular staining for signature transcription factors. This revealed that CD5^+^CD7^+^ lung ILCs were distinct from ILC2s and ILC3s, because they lacked expression of the lineage-defining transcription factors GATA3 and RORγt (**Figure 2B**). Consistent with containing cells with an ILC1 surface phenotype (CD117^-^CRTH2^-^), the CD5^+^CD7^+^ ILC subset expressed T-BET, although less than NK cells (**Figure 2B**). These data suggested that human CD5^+^CD7^+^ ILCs in the lung contained some mature ILC1s. To explore this possibility, we performed intracellular cytokine staining after stimulation with PMA and ionomycin *in vitro*. These experiments confirmed that a fraction of CD5^+^CD7^+^ ILCs produced the ILC1 signature cytokine IFNγ (**Figures 2C, D**). In contrast, the frequency of IL-22-/IL17A-expressing ILC3s was very low in the lungs of HSPC-engrafted MISTRG mice (**Figures 2C, D**), consistent with a lack of NKp44^+^ ILCs (**Figure 2E**) that normally produce IL-22 (51, 52). These results are in accordance with previous data, reporting that NKp44^+^ ILC3s are rare in the human lung at steady state (30). CD5^+^CD7^+^ ILCs also contained some mature ILC2s producing IL-13, but their frequency was lower than within the CD5^-^CD7^+^ ILC population (**Figures 2C, D**). Combined, these data support the notion that CD5^+^CD7^+^ ILCs consisted not only of mature ILC1s, but also of functionally immature ILCs that do not produce effector cytokines.

### Human CD5^+^CD7^+^ ILCs Have an Immature Surface Phenotype and Are Actively Dividing

**Figure 3:**
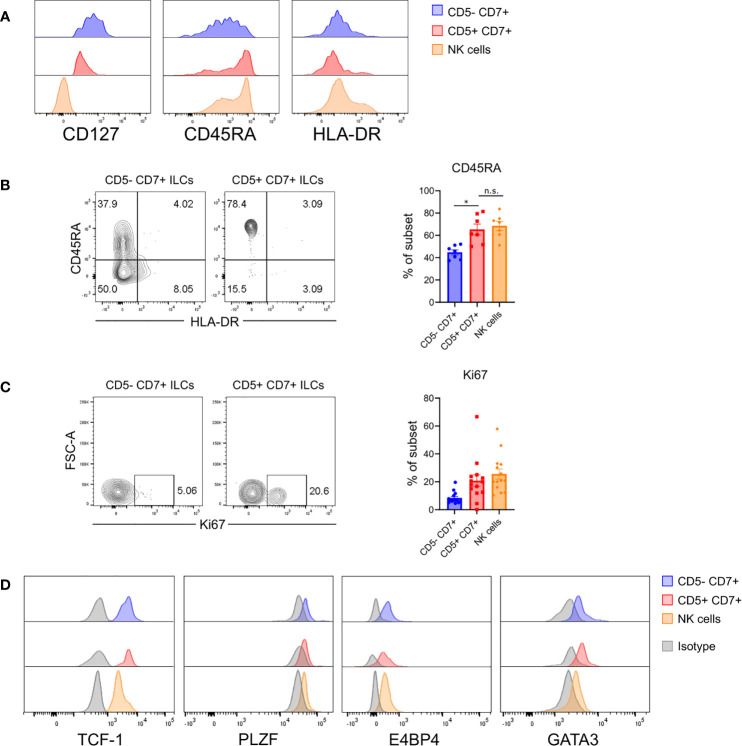
Human CD5^+^CD7^+^ ILCs have an immature surface phenotype and are actively dividing. **(A)** Surface expression of CD127, CD45RA, and HLA-DR on human CD5^-^CD7^+^ ILCs, CD5^+^CD7^+^ ILCs, and NK cells in the lung of HSPC-engrafted MISTRG mice. ILCs were gated as human CD45^+^Lin^-^CD3^-^TCRαβ^-^CD94^-^CD127^+^ cells and NK cells were gated as human CD45^+^Lin^-^CD3^-^TCRαβ^-^CD94^+^CD127^-^ cells as in **Figure 1B**. **(B)** Frequencies of CD45RA^+^ cells among CD5^-^CD7^+^ ILCs, CD5^+^CD7^+^ ILCs, and NK cells from the lung of HSPC-engrafted MISTRG mice (n = 7). n.s., not significant; *P < 0.05 by one-way ANOVA, Tukey’s post-test. **(C)** Intracellular expression of the proliferation marker Ki67 in CD5^-^CD7^+^ ILCs, CD5^+^CD7^+^ ILCs, and NK cells from the lung of HSPC-engrafted MISTRG mice and frequencies of Ki67^+^ cells among each subset (n = 14). **(D)** Intracellular expression of transcription factors (TCF-1, PLZF, E4BP4, GATA3) in human CD5^-^CD7^+^ ILCs, CD5^+^CD7^+^ ILCs, and NK cells isolated from the spleen of HSPC-engrafted MISTRG mice. ILCs were gated as human CD45^+^Lin^-^CD3^-^TCRαβ^-^CD127^+^ cells and NK cells were gated as human CD45^+^Lin^-^CD3^-^TCRαβ^-^CD127^-^CD56^+^ cells. Matched isotype antibodies were used as controls. Data represent mean ± SEM and are representative of at least two independent experiments.

The above findings raised the possibility that CD5^+^CD7^+^ ILCs represented an immature ILC population. To further characterize this subset, we examined surface markers related to ILC maturation status. Flow cytometry showed that CD5^+^CD7^+^ ILCs in the lung of HSPC-engrafted MISTRG mice had intermediate CD127 expression, but did not express the mature ILC markers HLA-DR (**Figures 3A, B**) and NKp44 (**Figure 2E**). Instead, CD5^+^CD7^+^ ILCs mostly expressed CD45RA (**Figures 3A, B**), a surface protein found on naïve T cells as well as ILCPs and resting ILCs (24, 33, 53). These results indicated that CD5^+^CD7^+^ ILCs consisted of immature or naïve-like cells. To further explore this possibility, we investigated whether CD5^+^CD7^+^ ILCs from the lung of HSPC-engrafted MISTRG mice can proliferate. Staining for the proliferation marker Ki67 demonstrated that this subset contains actively dividing cells in steady state (**Figure 3C**). Finally, we determined the expression of transcription factors regulating ILC development (54). Human CD5^+^CD7^+^ ILCs from the spleen of HSPC-engrafted MISTRG mice expressed intracellular TCF-1 and variable amounts of PLZF and E4BP4 protein, like their CD5^-^CD7^+^ counterparts (**Figure 3D**). TCF-1 is associated with “stemness” and known to be expressed by naïve T cells, T memory stem cells, as well as circulating CD117^+^ ILCPs (24). Furthermore, TCF-1 is expressed by mouse ILCPs (16). This further supports the notion that CD5^+^CD7^+^ ILCs have features of immature ILCs. In contrast to the lung (**Figure 2B**), human CD5^+^CD7^+^ ILCs, as well as NK cells and CD5^-^CD7^+^ ILCs from the spleen of HSPC-engrafted MISTRG mice expressed some GATA3 (**Figure 3D**). While ILC2s have the highest GATA3 expression, GATA3 is also known to be expressed by circulating CD117^+^ ILCPs (24, 34), consistent with its general role in ILC development (54). Taken together with our results above, we conclude that CD5^+^CD7^+^ ILCs contain actively dividing cells with features of immature ILCs.

### Human CD5^+^CD7^+^ ILCs Respond to Polarizing Cytokines *In Vitro*

**Figure 4:**
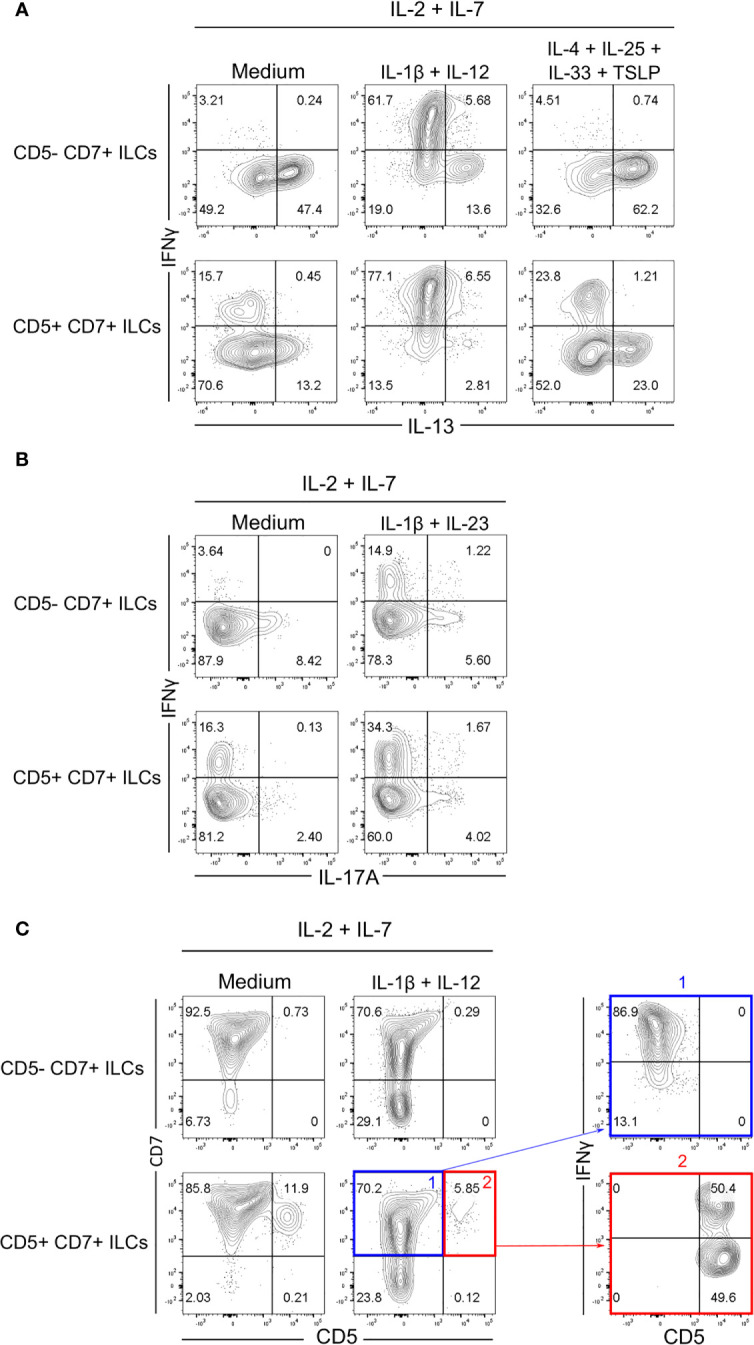
Human CD5^+^CD7^+^ ILCs respond to polarizing cytokines *in vitro.* **(A, B)** Flow cytometry analysis of intracellular IFNγ, IL-13 **(A)**, and IL-17A **(B)** expression by CD5^-^CD7^+^ ILCs (top row) and CD5^+^CD7^+^ ILCs (bottom row) one week after co-culture with OP9-DL1 cells in the presence of the indicated polarizing cytokines. CD5^-^CD7^+^ and CD5^+^CD7^+^ ILCs were purified from human peripheral blood by cell sorting. Cells were gated as in **Supplementary Figure 3A**. Non-stimulated controls are shown in **Supplementary Figure 3B**. **(C)** Cell surface expression of CD5 and CD7 on purified CD5^-^CD7^+^ ILCs (top row) and CD5^+^CD7^+^ ILCs (bottom row) one week after co-culture with OP9-DL1 cells and IL-2+IL-7 only or with OP9-DL1 cells and IL-2+IL-7+IL1β+IL-12. Dot plots on the right show IFNγ production by CD5^+^CD7^+^ ILCs that have down-modulated (blue) or maintained (red) CD5 surface expression. Data are representative of at least two independent experiments.

To further examine the differentiation potential and effector function of CD5^+^CD7^+^ ILCs, we purified both CD5^+^CD7^+^ and CD5^-^CD7^+^ ILC subsets from human CD127^+^Lin^-^CD3^-^TCRαβ^-^CD94^-^ ILCs isolated from adult peripheral blood and co-cultured them with OP9-DL1 cells *in vitro* with various lineage-polarizing cytokines. CD5^-^CD7^+^ ILCs from human blood are known to contain both mature ILCs (mainly ILC2s) and CD117^+^ ILCPs that are able to differentiate into all types of ILCs (24). Accordingly, purified CD5^-^CD7^+^ ILCs produced IL-13 after *in-vitro* culture without any lineage-skewing condition (IL-2+IL-7 only), which was further increased in the presence of ILC2-polarizing cytokines (IL-4+IL-25+IL-33+TSLP) (**Figure 4A** and **Supplementary Figure 3A**). The CD5^-^CD7^+^ subsets also gave rise to IFNγ-producing ILC1s (or NK cells) when cultured in ILC1-polarizing cytokines (IL-1β+IL-12) (**Figure 4A**) and, to a lesser extent, to IL-17A-producing ILC3s when ILC3 polarizing cytokines (IL-1β+IL-23) were present (**Figure 4B**). On the other hand, CD5^+^CD7^+^ ILCs purified from blood contained some mature ILC1s and ILC2s as demonstrated by spontaneous IFNγ and IL-13 production in neutral culture conditions (IL-2+IL-7 only) (**Figure 4A**). In lineage-skewing conditions, the frequency of effector cytokine-producing ILCs derived from CD5^+^CD7^+^ ILCs increased, with a predominance of IFNγ^+^ cells (**Figures 4A, B** and **Supplementary Figure 3**). These results indicated that the CD5^+^CD7^+^ ILC population either contained pre-existing mature ILCs that expanded during the *in-vitro* culture and/or immature ILCs that acquired the ability to produce effector cytokines in response to polarizing signals. Moreover, we observed that purified CD5^+^CD7^+^ ILCs largely lost CD5 surface expression *in vitro*, irrespective of the culture conditions (**Figure 4C**). Further analysis revealed that IFNγ-producing ILC1s (or NK cells) were mainly enriched within purified CD5^+^CD7^+^ ILCs that had downregulated CD5 from their cell surface after *in-vitro* culture (**Figure 4C**). In contrast, purified CD5^+^CD7^+^ ILCs that had retained CD5 expression consisted of IFNγ^+^ ILC1s (or NK cells) as well as cells that did not produce IFNγ, even when ILC1-polarizing cytokines were present in the culture (**Figure 4C**). Taken together, we demonstrate that CD5^+^CD7^+^ ILCs acquire effector function after downregulating CD5 from their cell surface.

### CD5^+^CD7^+^ ILCs Mainly Reside in the Lung Vasculature

**Figure 5:**
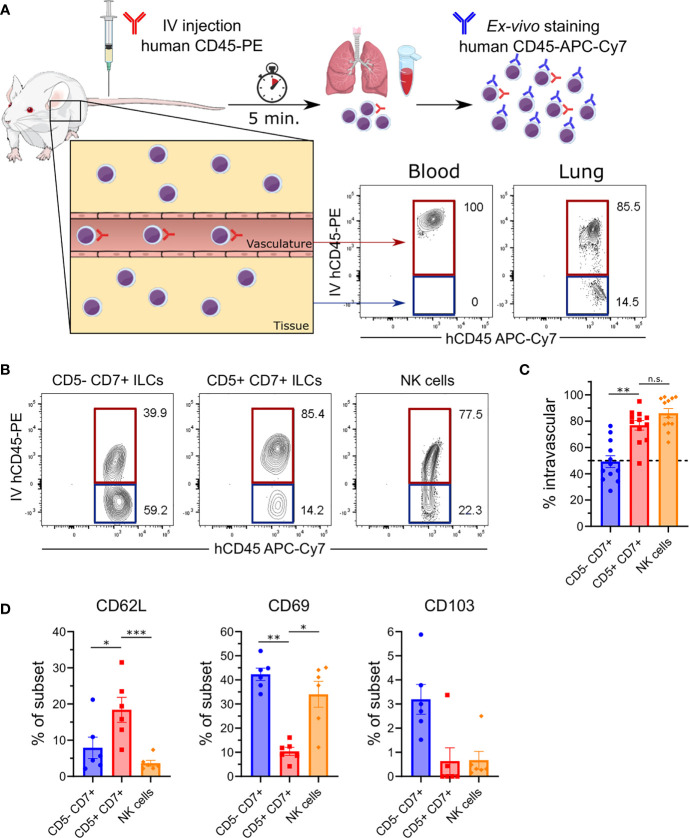
CD5^+^CD7^+^ ILCs mainly reside in the lung vasculature. **(A)** Intravascular labeling of human CD45^+^ hematopoietic cells in MISTRG mice engrafted with CD34^+^ HSPCs by intravenous (IV) injection of anti-human CD45-PE antibody. Human cells in the vasculature (highlighted in red) are stained by both the IV-injected anti-CD45 antibody (hCD45-PE) and by the *ex-vivo* anti-CD45 antibody (hCD45-APC-Cy7). Cells residing in the tissue (highlighted in blue) are only stained with the *ex-vivo* hCD45-APC-Cy7 antibody. **(B)** Flow cytometry analysis of intravascular (red) and extravascular (blue) CD5^-^CD7^+^ ILCs, CD5^+^CD7^+^ ILCs, and NK cells in the lung of HSPC-engrafted MISTRG mice. ILCs were gated as human CD45^+^Lin^-^CD3^-^TCRαβ^-^CD94^-^CD127^+^ cells and NK cells were gated as human CD45^+^Lin^-^CD3^-^TCRαβ^-^CD94^+^CD127^-^ cells as in **Figure 1B**. **(C)** Frequencies of intravascular (IV-hCD45-PE^+^) human CD5^-^CD7^+^ ILCs, CD5^-^CD7^+^ ILCs, and NK cells in the lung (n = 12) as determined in **(B)**. **(D)** Frequency of CD62L, CD69, and CD103 expression by human lung CD5^-^CD7^+^ ILCs, CD5^-^CD7^+^ ILCs, and NK cells from HSPC-engrafted MISTRG mice (n = 6). n.s., not significant; *P < 0.05, **P < 0.01, ***P < 0.001 by one-way ANOVA, Tukey’s post-test. Data represent mean ± SEM and are representative of at least two independent experiments. **(A)** was adapted from Mind the Graph.

Our observation that human CD5^+^CD7^+^ ILCs were abundant in peripheral blood (**Figure 1E**) suggested that they resided in a different anatomical compartment than conventional CD5^-^CD7^+^ ILCs. To investigate whether CD5^+^ and CD5^-^ ILC subsets occupied different niches in the lung, we performed intravascular cell labeling by intravenously (IV) injecting HSPC-engrafted MISTRG mice with a phycoerythrin (PE)-conjugated antibody against human CD45 (**Figure 5A**). This technique allowed us to distinguish cells within the lung vasculature (stained by IV CD45-PE antibody) from cells within lung tissue, i.e. outside of lung blood vessels, that were not stained by IV CD45-PE antibody (22). As expected, all CD45^+^ hematopoietic cells in peripheral blood were labeled by the IV CD45-PE antibody, whereas bronchoalveolar lavage (BAL) cells outside of the lung vasculature were not stained (**Supplementary Figure 4**). Flow cytometric analysis of lung tissue after intravascular antibody labeling revealed differences in the intravascular and extravascular distribution of human ILCs in MISTRG mice. Specifically, we found that conventional CD5^-^CD7^+^ ILCs resided in both the intravascular and extravascular compartment of the lung (**Figures 5B, C**). In contrast, CD5^+^CD7^+^ ILCs mostly had an intravascular localization in the lung, similar to NK cells (**Figures 5B, C**).

To further explore the migratory features of CD5^+^CD7^+^ ILCs, we examined the expression of cell surface receptors that regulate ILC trafficking and tissue residency (9, 10). Compared to the other ILC subsets, very few CD5^+^CD7^+^ ILCs expressed the tissue residency markers CD69 and CD103 on their cell surface (**Figure 5D**), consistent with their intravascular localization in the lung (**Figures 5B, C**). Instead, CD5^+^CD7^+^ ILCs more frequently expressed CD62L surface protein (**Figure 5D**), which is known to mediate the homing of naïve T lymphocytes to secondary lymphoid organs. Overall, these findings demonstrate that CD5^+^CD7^+^ ILCs have a unique anatomical location within the lung and that they share an intravascular niche with NK cells.

### CD5^+^CD7^+^ ILCs Have a Distinct Ontogeny Compared to CD5^-^CD7^+^ ILCs

**Figure 6:**
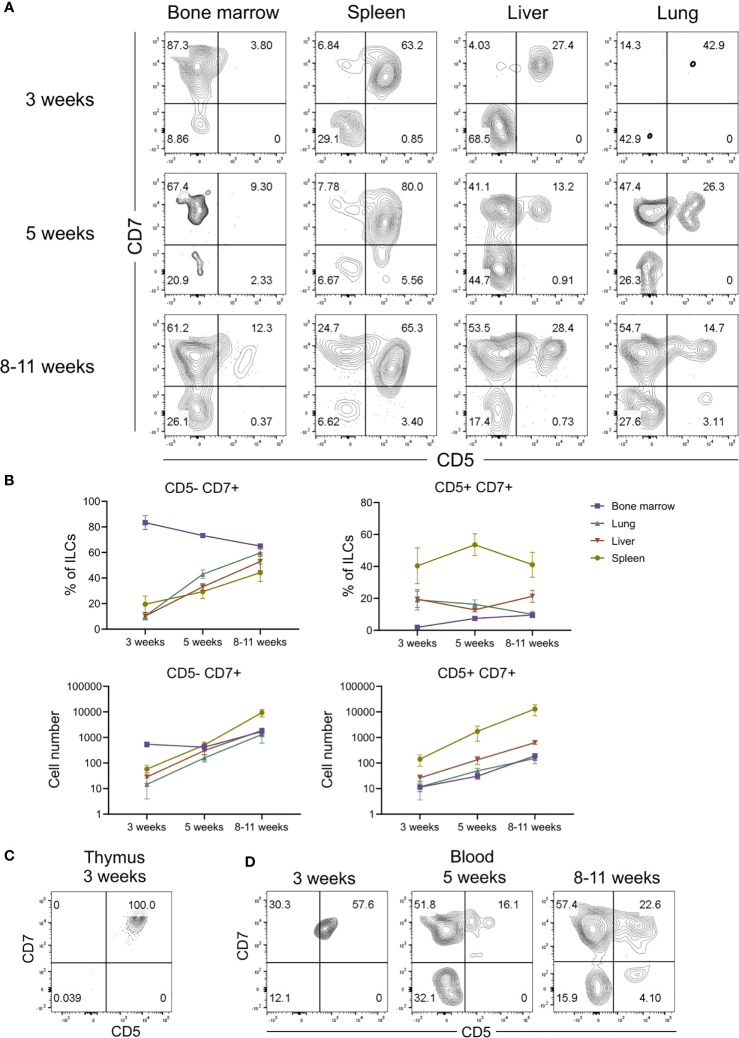
CD5^+^CD7^+^ ILCs have a distinct ontogeny compared to CD5^-^CD7^+^ ILCs. **(A)** Flow cytometry analysis of CD5/CD7-expressing human ILCs in bone marrow, spleen, liver, and lung at 3 weeks, 5 weeks and 8-10 weeks after transplantation of MISTRG mice with human CD34^+^ HSPCs. ILCs were gated as human CD45^+^Lin^-^CD3^-^TCRαβ^-^CD94^-^CD127^+^ cells. **(B)** Frequency and numbers of CD5^-^CD7^+^ and CD5^-^CD7^+^ ILCs in bone marrow, spleen, liver, and lung of MISTRG mice at 3 weeks, 5 weeks and 8-10 weeks post-engraftment with HSPCs (n = 6-9). **(C)** Flow cytometry analysis of human CD5^+^CD7^+^ ILCs in the thymus of MISTRG mice at 3 weeks after transplantation with human CD34^+^ HSPCs. **(D)** Flow cytometry analysis of human CD5^+^CD7^+^ ILCs in the blood of MISTRG mice at 3 weeks, 5 weeks and 8-10 weeks after transplantation with human CD34^+^ HSPCs. Data represent mean ± SEM and are representative for at least two independent experiments.

**Figure 7:**
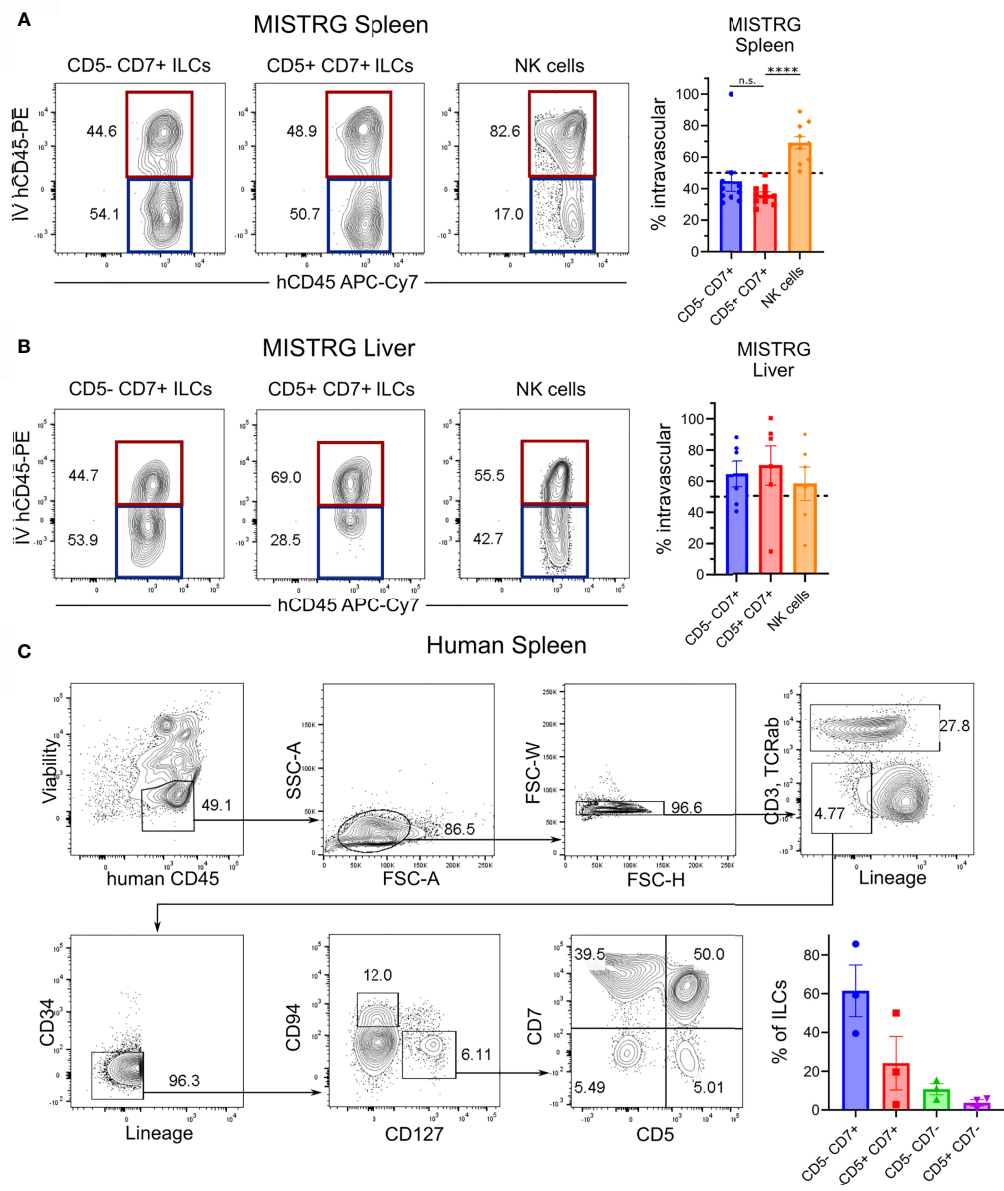
Human CD5^+^CD7^+^ ILCs reside in the spleen and liver. **(A, B)** Flow cytometry analysis of intravascular (red) and extravascular (blue) CD5^-^CD7^+^ ILCs, CD5^+^CD7^+^ ILCs, and NK cells in the spleen **(A)** and liver **(B)** of HSPC-engrafted MISTRG mice. Frequencies of intravascular (IV-hCD45-PE^+^) spleen and liver ILC subsets and NK cells (n = 6-10) are shown on the right. n.s., not significant; ****P < 0.0001 by one-way ANOVA, Tukey’s post-test. **(C)** Frequencies of CD5^-^CD7^+^ and CD5^+^CD7^+^ ILCs in human spleen (n = 3). ILCs were gated as shown as viable human CD45^+^CD3^-^TCRαβ^-^CD94^-^CD127^+^ cells that were negative for lineage markers (CD11c, CD14, CD19, CD123, FceRI) and CD34. Data represent mean ± SEM and are representative of three independent experiments.

In adult hosts, conventional ILCs develop from a common lymphoid progenitor in the bone marrow and complete their differentiation into mature ILCs after migration into various tissues (54–57). The finding that CD5^+^CD7^+^ ILCs inhabited a different anatomical niche than conventional CD5^-^CD7^+^ ILCs raised the possibility that CD5^+^CD7^+^ ILCs have a distinct developmental origin. Moreover, the expression of CD62L by CD5^+^CD7^+^ ILCs (**Figure 5D**) suggested that they home to secondary lymphoid organs, such as the spleen. To determine the origin of CD5^+^CD7^+^ ILCs, we examined the reconstitution of the human ILC compartment in bone marrow, spleen, liver, and lung of MISTRG mice at different time points after transplantation with human CD34^+^ HSPCs. Human T cells and NK cells greatly expanded in all organs between 3 and 5 weeks post-transplantation (**Supplementary Figure 5**). Human ILCs could already be detected in bone marrow, spleen, and liver 3 weeks post-transplantation, whereas there were few ILCs in the lung at this early time point (**Figure 6A** and **Supplementary Figure 5**). As expected, conventional CD5^-^CD7^+^ ILCs were present in the bone marrow (**Figures 6A, B**), their known site of development. In contrast, CD5^+^CD7^+^ ILCs mainly resided in the liver and spleen already 3 weeks after HSPC transplantation (**Figures 6A, B**), suggesting that they originated from CD34^+^ HSPCs or local precursors in these organs. We also considered the possibility that human CD5^+^CD7^+^ ILCs in MISTRG mice had a thymic origin before migrating to the spleen and liver. Flow cytometry revealed the presence of CD34^-^Lin^-^CD5^+^CD7^+^ cells lacking CD3 and TCRab surface expression in the thymus of MISTRG mice 3 weeks after transplantation (**Figure 6C**). This result is consistent with previous data showing that CD5^+^CD7^+^ ILCs are present in the human post-natal thymus (34) and raised the possibility that CD5^+^ ILCs originate from precursors in the thymus. The finding that reconstitution of human CD5^+^CD7^+^ ILCs in the lung occurred later than in the other organs suggested that lung ILCs were derived from cells from the thymus, liver and/or spleen that had migrated to the lung *via* the blood. In support of this possibility, circulating CD5^+^CD7^+^ ILCs were present in MISTRG mice already at 3 weeks after transplantation with human CD34^+^ HSPCs (**Figure 6D**). Furthermore, human CD5^+^CD7^+^ ILCs were found in both the extravascular and the intravascular compartment of the spleen and liver of HSPC-engrafted MISTRG mice (**Figures 7A, B**). This further supported the notion that CD5^+^CD7^+^ ILCs derived from intrahepatically injected CD34^+^ HSPCs took up residence in the spleen and liver of MISTRG mice and subsequently gained access to the local vasculature (spleen red pulp, liver sinusoids) and systemic circulation. Importantly, CD5^+^CD7^+^ ILCs were also present in the spleen of human organ donors (**Figure 7C**), confirming the physiological relevance of our findings. Collectively, these results support the notion that CD5^+^CD7^+^ ILCs have a distinct origin and ontogeny than their CD5^-^CD7^+^ counterparts.

## Discussion

ILCs are essential for rapid immune responses and organ homeostasis. However, the ontogeny and migration of human ILCs is difficult to study in blood and tissue samples *ex vivo*. Accordingly, the developmental and migratory pathways of human ILCs remain poorly defined. To overcome this limitation, we employed a humanized mouse model to investigate human ILC migration within a surrounding tissue microenvironment *in vivo*. Using this experimental system, we identified a human CD5-expressing ILC population in the lung that has a unique ontogeny and migratory behavior, distinct from that of conventional CD5^-^ ILCs. CD5^+^CD7^+^ ILCs were located within the vasculature and may represent circulating precursors of mature human ILCs. Furthermore, vascular CD7^+^CD5^+^ ILCs had a different developmental path than tissue ILCs, first appearing in the spleen rather than in the bone marrow after transplantation of MISTRG mice with human CD34^+^ HSPCs **(** **Supplementary Figure 6** **)**. A few reports have studied human CD5^+^ ILCs (34, 39), but CD5^+^ ILCs remain poorly characterized and the lack of a relevant *in-vivo* model to study them has been a major limitation. Beyond these previous reports, our study provides new knowledge on the ontogeny, anatomical localization, and migration of human CD5^+^ ILCs.

The CD5^+^CD7^+^ ILC population occupied the intravascular niche in the lung and other highly vascularized organs, such as the liver and spleen, after birth, mostly staying within the circulation in steady state, unlike conventional ILCs that migrate into the lung and become tissue-resident. Furthermore, in contrast to conventional CD5^-^CD7^+^ ILCs that develop in the bone marrow, CD5^+^CD7^+^ ILCs appeared first in thymus, liver, and spleen after transplantation of MISTRG mice with CD34^+^ HSPCs. Therefore, the timing and pattern of tissue reconstitution differed between CD5^+^CD7^+^ ILCs and CD5^-^CD7^+^ ILCs, which suggests that CD5^+^CD7^+^ ILCs may develop along an alternative pathway, distinct from that of bone marrow-derived CD5^-^CD7^+^ ILCs. Specifically, CD5^+^CD7^+^ ILCs could arise locally from intrahepatically injected CD34^+^ HSPCs before disseminating *via* the circulation. This notion is supported by a recent study in mice demonstrating local differentiation of mouse ILC1s from hematopoietic stem cells in the liver (58). Alternatively, it is possible that CD5^+^CD7^+^ ILCs in MISTRG mice develop from local precursors in the spleen, consistent with CD62L surface expression by CD5^+^CD7^+^ ILCs. Moreover, studies in humanized mice indicate that the spleen environment may favor human ILC1 differentiation (59). The expression of T cell-related molecules suggests that CD5^-^CD7^+^ ILCs may also have a thymic origin, possibly representing ILCs with a failed T cell program. Finally, we cannot exclude the possibility that CD5^+^CD7^+^ ILCs originate from the bone marrow and then migrate to other organs, such as liver and spleen, although this scenario seems less likely based on our data. Overall, our results suggest that there are non-redundant pathways of human ILC development and that distinct developmental paths may relate to specific anatomical niches occupied by ILCs.

As their innate counterparts, ILCs share several features with T cells and they express T cell-related molecules, such as the surface protein CD5, which has important roles in T cell development and regulation (28, 29). Earlier studies demonstrated that ILC1s can express CD5 (33–38). Moreover, ILC populations expressing CD5 have been described as precursors to mature ILC2s and KIR^+^NKG2A^-^ NK cells (34, 39). Consistent with these reports, our study further demonstrates that CD5^+^ ILCs are heterogeneous and contain ILC populations other than mature ILC1s. We describe CD5^+^CD7^+^ ILCs with a surface phenotype (CD45RA^+^HLA-DR^-^CD62L^+^) that is associated with naïve T cells recirculating between lymphoid organs. Furthermore, these CD5^+^CD7^+^ ILCs were present in highly vascularized organs (lung, spleen, and liver) with a mainly intravascular localization and were able to produce signature cytokines of mature ILC1s, ILC2s, and to a lesser extent ILC3s under polarizing conditions *in vitro*. We observed that these CD5^+^CD7^+^ ILCs downregulated CD5 from the cell surface upon acquiring effector function. A similar finding was reported by the Spits’ lab for CD5^+^ ILC2s that develop into cytokine-secreting CD5^-^ ILC2s (34). It is therefore possible that CD5 inhibits the effector function of CD5^+^CD7^+^ ILCs.

The exact lineage identity of CD5^+^ ILCs is still controversial. CD5^+^ ILCs are related to T cells as shown by the expression of T cell signature genes, intracellular CD3 protein, and the presence of *TCR* gene rearrangements (34, 35, 38, 39, 50, 60, 61). However, these *TCR* gene rearrangements likely result in nonfunctional TCR protein expression. This idea is supported by a recent study, demonstrating non-productive *TCR* gene rearrangements in mouse ILC2s (62). Moreover, in contrast to T cells, ILC1s expressing CD5 are present in the blood of humans with *RAG1* deficiency (37). Overall, this indicates that expression of T cell-associated surface markers, such as CD5, and intracellular CD3 expression is *per se* not sufficient to define T cell identity. CD5^+^ ILCs may represent immature ILCs with precursor activity or failed T cells with a nonfunctional TCR that underwent reprogramming into ILCs. Therefore, conclusively defining the exact lineage identity of CD5^+^ ILCs is an important area of future study.

Their intravascular localization within the lung shows that CD5^+^ ILCs are distinct from conventional CD5^-^ ILCs that mostly reside outside of the vasculature in the lung. Furthermore, their migratory features are likely linked to their function. Although their residence time within the lung vasculature is unknown, it is possible that CD5^+^CD7^+^ ILCs adhere to the endothelium within lung capillaries and become a marginated pool of cells. Therefore, CD5^+^CD7^+^ ILCs could act as sentinels, patrolling the body *via* the blood vessels, and migrate “on-demand” into tissue during infection and other immune challenges. This might be especially relevant in the lung (63), a highly vascularized organ that is the first to receive the full cardiac output. CD5^+^CD7^+^ ILCs would be well-suited for this task, because they constitute a mixture of mature ILC1s, ready to contribute to type 1 immune responses, and immature cells that could produce effector cytokines when exposed to polarizing cytokines within the inflamed lung. Consistent with this possibility, circulating ILCs are reduced in respiratory infections, such as COVID-19 (64), suggesting their active recruitment into the lung. Therefore, vascular CD5^+^CD7^+^ ILCs may perform similar functions to blood monocytes that patrol blood vessels and gain access to tissue niches during altered organ homeostasis, when they differentiate into tissue-resident macrophages to support host defense against infection (65).

Type 1 immune responses are directed against viruses and intracellular bacteria and put the host in a state of “anticipatory alert”, associated with heightened immune surveillance and tissue resistance to infection (66). ILCs involved in type 1 immunity are mainly IFNγ-secreting ILC1s/NK cells and cytotoxic NK cells, which both are mainly intravascular. Cytotoxic NK cells are kept in check by the expression of inhibitory NK cell receptors. We speculate that CD5 performs a similar role to inhibit the activity CD5^+^CD7^+^ ILC1s, in analogy to the inhibitory function of CD5 in T cells. This would allow the host to locally compartmentalize immune responses in order to avoid immunopathology and improper systemic immune activation within the vasculature. Therefore, future studies should investigate the role of CD5^+^ ILCs in pulmonary infections and various inflammatory lung diseases in humans.

### Limitations of the Study

MISTRG mice allow the investigation of the human immune system in a small animal model and are therefore suitable to study human ILCs *in vivo*. Limitations of this and other humanized mouse models include the absence of human fetal hematopoiesis because some ILCs, e.g. in the intestine, develop from fetal progenitors (67, 68). Another limitation is the lack of fully developed lymph nodes, which might affect ILC development and trafficking. Furthermore, human ILCs interact with mouse epithelial, stromal, and endothelial cells in HSPC-engrafted MISTRG mice as well as in other humanized mouse models (24, 48, 49, 69–71). However, the human cytokines in MISTRG mice are sufficient to support human ILC-poiesis from HSPCs. Moreover, human ILCs migrate into tissues in MISTRG mice, leading to the distribution of the different ILC subsets into distinct vascular and tissue compartments. Overall, despite some shortcomings, our experimental system provides important information on the *in-vivo* biology of human ILCs, complementary to *ex-vivo* studies of ILCs from human blood and tissues.

Previous studies have demonstrated that CD5^+^ ILCs from human blood contain ILC2 and NK cell precursors (34, 39). We observed that, after bulk culture, purified CD5^+^ ILCs produced ILC1, ILC2, and, to a lesser extent, ILC3 effector cytokines *in vitro*. However, we cannot exclude the possibility that this is due to the outgrowth of pre-existing mature ILCs. Therefore, future studies, using single-cell ILC differentiation assays, are needed to unequivocally determine whether CD5^+^ ILCs contain multi-potent ILCPs.

## Data Availability Statement

The original contributions presented in the study are included in the article/**Supplementary Material**. Further inquiries can be directed to the corresponding author.

## Ethics Statement

The studies involving human participants were reviewed and approved by the Ethical Review Boards at Karolinska Institutet (#2006/229-31/3, 2015/1368-31/4, 2015/2122-32, 2016/1415-32, 2019-05016). The patients/participants provided their written informed consent to participate in this study. The animal study was reviewed and approved by the Linköping Animal Experimentation Ethics Committee (#29-15, 03127-2020).

## Author Contributions

AA designed, performed, and analyzed most experiments and wrote the paper. YG and EE designed, performed, and analyzed some experiments. NS helped with mouse experiments. DB, AK, CJ, NM, and JM provided human lung cells. TW conceived and supervised the study, acquired funding, designed, and analyzed experiments, and wrote the paper. All authors contributed to the article and approved the submitted version.

## Funding

This work was supported by a faculty-funded career position at Karolinska Institutet (2-1060/2018), a KID grant from Karolinska Institutet (2018-00846), a Junior Investigator and Junior Project Research Grant from the Center for Innovative Medicine (CIMED) financed by Region Stockholm (2-538/2014; 20190152), as well as Project Grants from the Swedish Research Council (2015-02413; 2019-01099), the Swedish Heart-Lung Foundation (20190198), and Petrus och Augusta Hedlunds Stiftelse (M-2021-1568) to TW.

## Conflict of Interest

The authors declare that the research was conducted in the absence of any commercial or financial relationships that could be construed as a potential conflict of interest.

## Publisher’s Note

All claims expressed in this article are solely those of the authors and do not necessarily represent those of their affiliated organizations, or those of the publisher, the editors and the reviewers. Any product that may be evaluated in this article, or claim that may be made by its manufacturer, is not guaranteed or endorsed by the publisher.

## Supplementary Material

Supplementary Table 1 List of antibodies used for flow cytometry.

## Abbreviations

HSPCs, human hematopoietic stem and progenitor cells; IFNg, interferon gamma; IL, interleukin; ILCs, innate lymphoid cells; ILCPs, innate lymphoid cell precursors; IV, intravenous; Lin, lineage; NK, natural killer.
