# Supplementary material for: CD5 Surface Expression Marks Intravascular Human Innate Lymphoid Cells That Have a Distinct Ontogeny and Migrate to the Lung
Source: Front Immunol. 2021 Nov 18;12:752104. doi: 10.3389/fimmu.2021.752104 (PMC8640955; doi:10.3389/fimmu.2021.752104)
Supplement: Supplementary Table 1 — List of antibodies used for flow cytometry. [file Table_1.docx]

**Supplementary Table 1. List of antibodies used for flow cytometry**

| Antibodies | Source | Catalog number | Amount or dilution in 100 μl |
| --- | --- | --- | --- |
| Surface phenotype staining |  |  |  |
| Anti-human CD3 BV650 (clone SK7) | Biolegend | 563999 | 3 μl |
| Anti-human CD3 BV650 (clone OKT3) | Biolegend | 317324 | 4 μl |
| Anti-human CD3 BV650 (clone UCTH1) | Biolegend | 300468 | 3 μl |
| Anti-human CD5 BV605 (clone L17F12) | Biolegend | 364020 | 4 μl |
| Anti-human CD7 PE-CF594 (clone M-T701) | BD | 562541 | 3 μl |
| Anti-human CD11c biotin (clone Bu15) | Biolegend | 337232 | 1:200 |
| Anti-human CD14 biotin (clone M5E2) | Biolegend | 300112 | 1:50 |
| Anti-human CD19 biotin (clone HIB19) | Biolegend | 302204 | 1:200 |
| Anti-human CD34 biotin (clone 581) | BD | 564669 | 1:200 |
| Anti-human CD34 BUV395 (clone 581) | BD | 563778 | 2 μl |
| Anti-human CD45 APC-Cy7 (HI30) | Biolegend | 304014 | 4 μl |
| Anti-human CD45 PE-Cy5.5 (HI30) | Biolegend | 304010 | 4 μl |
| Anti-mouse CD45 AF700 (clone 30-F11) | Biolegend | 103128 | 1:400 |
| Anti-human CD45RA BUV737 (clone HI100) | BD | 564442 | 3 μl |
| Anti-human CD56 BV785 (clone 5.1H11) | Biolegend | 362550 | 5 μl |
| Anti-human CD62L PE-Cy7 (clone DREG56) | Biolegend | 304822 | 4 μl |
| Anti-human CD69 BV785 (clone FN50) | Biolegend | 310932 | 3 μl |
| Anti-human CD94 FITC (clone DX22) | Biolegend | 305504 | 3 μl |
| Anti-human CD103 BUV395 (clone Ber-ACT8) | BD | 564346 | 4 μl |
| Anti-human CD117 PC5.5 (clone 104D2D1) | Beckman Coulter | B96754 | 3 μl |
| Anti-human CD123 biotin (clone 6H6) | Biolegend | 306004 | 1:200 |
| Anti-human CD127 BV421 (clone A019D5) | Biolegend | 351310 | 5 μl |
| Anti-human CRTH2 APC (or CD294, clone BM16) | Biolegend | 350110 | 4 μl |
| Anti-human FcεRI biotin (clone AER-37 or CRA-1) | Biolegend | 334606 | 1:200 |
| Anti-human HLA-DR BUV395 (clone G46-6) | BD | 564040 | 3 μl |
| Anti-human NKp44 PE-Cy7 (or CD336, clone P44-8) | Biolegend | 325116 | 5 μl |
| Anti-human TCRαβ BV650 (clone IP26) | BD | 745226 | 4 μl |
| Intracellular CD3/TCR staining |  |  |  |
| Anti-human CD3 APC (clone SK7) | Biolegend | 344812 | 0.25 μl |
| Anti-human TCRαβ PE (clone IP26) | BD | 564728 | 1-1.5 μl |
| Transcription factor staining |  |  |  |
| Anti-human T-bet FITC (clone 4B10) | Biolegend | 644812 | 0.5 μl |
| Anti-human GATA3 AF647 (clone 16E10A23) | Biolegend | 653810 | 5 μl |
| Anti-human RORγt PE (clone Q21-559) | BD | 563081 | 2 μl |
| Anti-human TCF-1 AF488 (clone C63D9) | Cell Signaling | 6444 | 2 μl |
| Anti-human E4BP4 PE (clone MABA223) | ThermoFisher | 12-9812-42 | 4 μl |
| Anti-human PLZF PE-Cy7 (clone 9E12) | Invitrogen | 25-9322-80 | 5 μl |
| Intracellular cytokine staining |  |  |  |
| Anti-human IFNγ FITC (clone B27) | Biolegend | 506504 | 5 μl |
| Anti-human IL-17A APC (clone CZ8-23G1) | Miltenyi | 130-094-519 | 1:10 |
| Anti-human IL-13 PE (clone JES10-5A2) | BD | 554571 | 1:100 |
| Anti-human IL-22 PE-Cy7 (clone 22URTI) | eBioscience | 25-7229-42 | 5 μl |
| Isotype controls |  |  |  |
| Mouse IgG1 FITC | Biolegend | 400107 | Matched |
| Mouse IgG1 PE | BD | 554680 | Matched |
| Mouse IgG1 APC | Biolegend | 400122 | Matched |
| Mouse IgG1 PE-Cy7 | Biolegend | 400125 | Matched |
| Mouse IgG2b PE | Biolegend | 402203 | Matched |
|  |  |  |  |
| Mouse IgG2b APC | Biolegend | 400322 | Matched |
| Armenian hamster IgG1 PE-Cy7 | Biolegend | 400921 | Matched |
| Rabbit IgG AF488 | Cell Signalling | 2975 | Matched |
