# Supplementary material for: CD5 Surface Expression Marks Intravascular Human Innate Lymphoid Cells That Have a Distinct Ontogeny and Migrate to the Lung
Source: Front Immunol. 2021 Nov 18;12:752104. doi: 10.3389/fimmu.2021.752104 (PMC8640955; doi:10.3389/fimmu.2021.752104)
Supplement: Supplementary file 2 [file DataSheet_1.pdf]

Figure S1

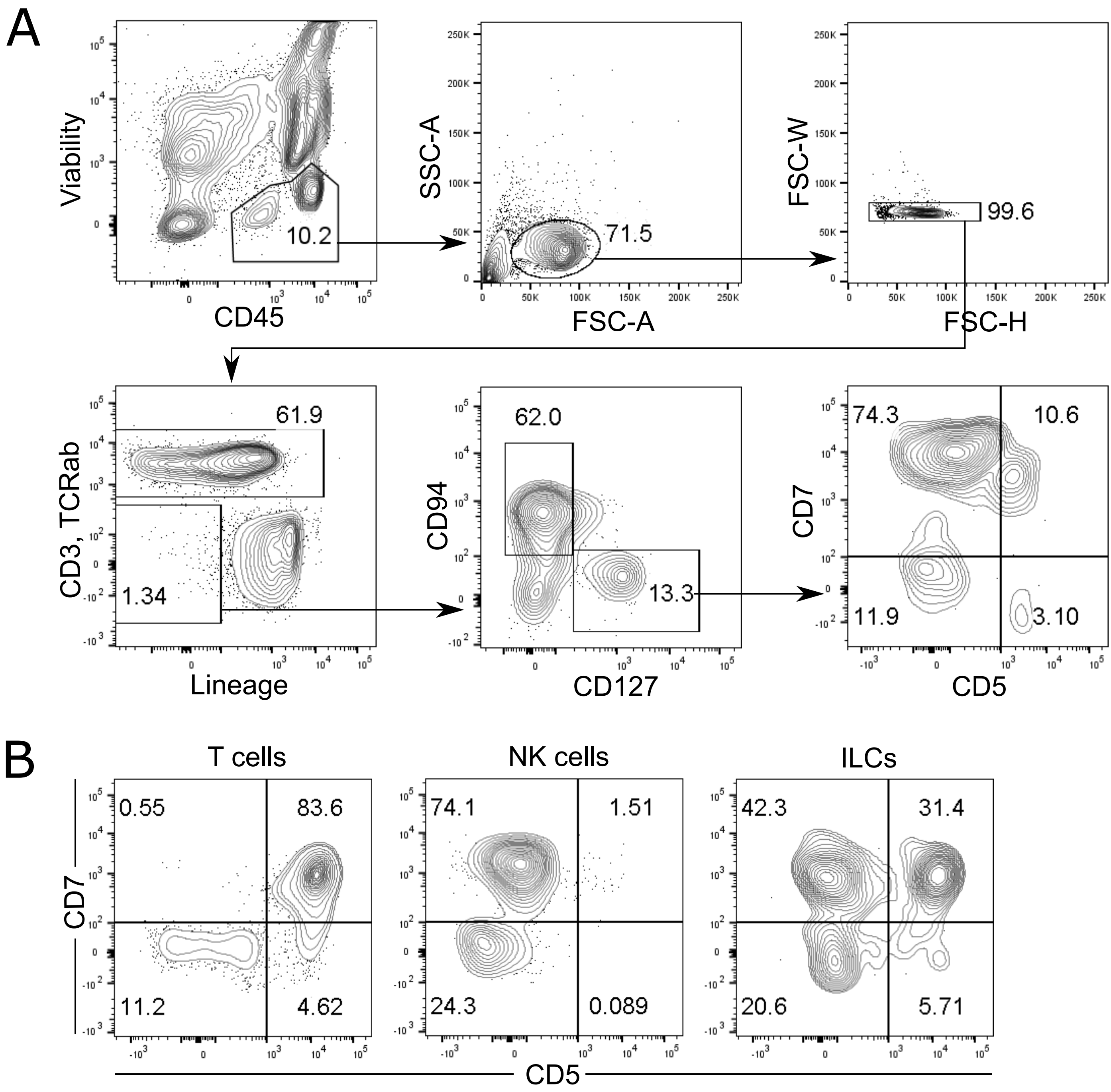

SUPPLEMENTARY FIGURE 1 CD5 is expressed by human ILCs, but not by human NK cells in the lung. **(A)** Gating strategy for the flow cytometric analysis of ILCs in human lung tissue from organ donors. Total ILCs were gated as viable human CD45 $^+$CD3$^-$TCRαβ$^-$CD94$^-$ CD127$^+$ cells that were negative for Lin eage markers (CD11c, CD14, CD19, CD34, CD123, FceRI). **(B)** Flow cytometry analysis of CD5 and CD7 cell surface expression on human T cells, NK cells and ILCs from the lung of HSPC-engrafted MISTRG mice. T cells (CD45$^+$ CD3$^+$TCRαβ$^+$), NK cells (CD45$^+$CD3$^-$TCRαβ$^-$CD94$^+$CD127$^-$), and ILCs (CD45$^+$Lineage$^-$ CD3$^-$TCRαβ$^-$CD94$^-$CD127$^+$) were gated as in Figure 1B. Data are repesentative of at least 3 experiments.

Figure S2

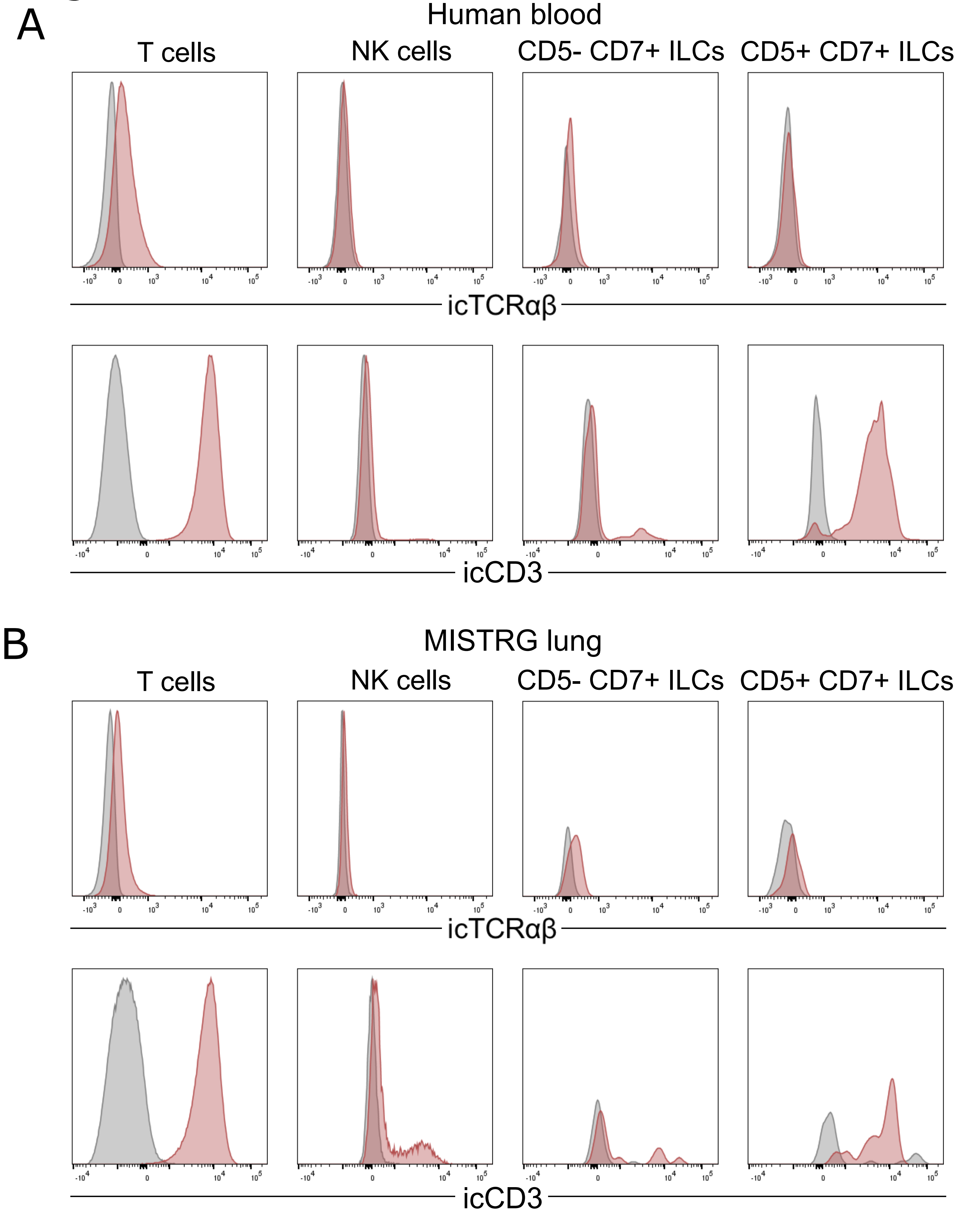

SUPPLEMENTARY FIGURE 2 CD5$^+$CD7$^+$ ILCs express intracellular CD3, but lack intracellular TCRαb protein. **(A and B)** Intracellular expression of CD3 and TCRαb in T cells, NK cells, CD5-CD7+ ILCs, and CD5$^+$CD7$^+$ ILCs from human peripheral blood (A) or from the lung of HSPC-engrafted MISTRG mice (B). T cells (CD45$^+$CD3$^+$TCRαβ$^+$), NK cells (CD45$^+$CD3$^-$TCRαβ$^-$CD94$^+$CD127$^-$), and ILCs (CD45$^+$Lineage$^-$CD3$^-$TCRαβ$^-$CD94$^-$CD127$^+$) were gated as in Figure 1B. Matched isotype antibodies were used as controls. Data are representative of at least two experiments.

Figure S3

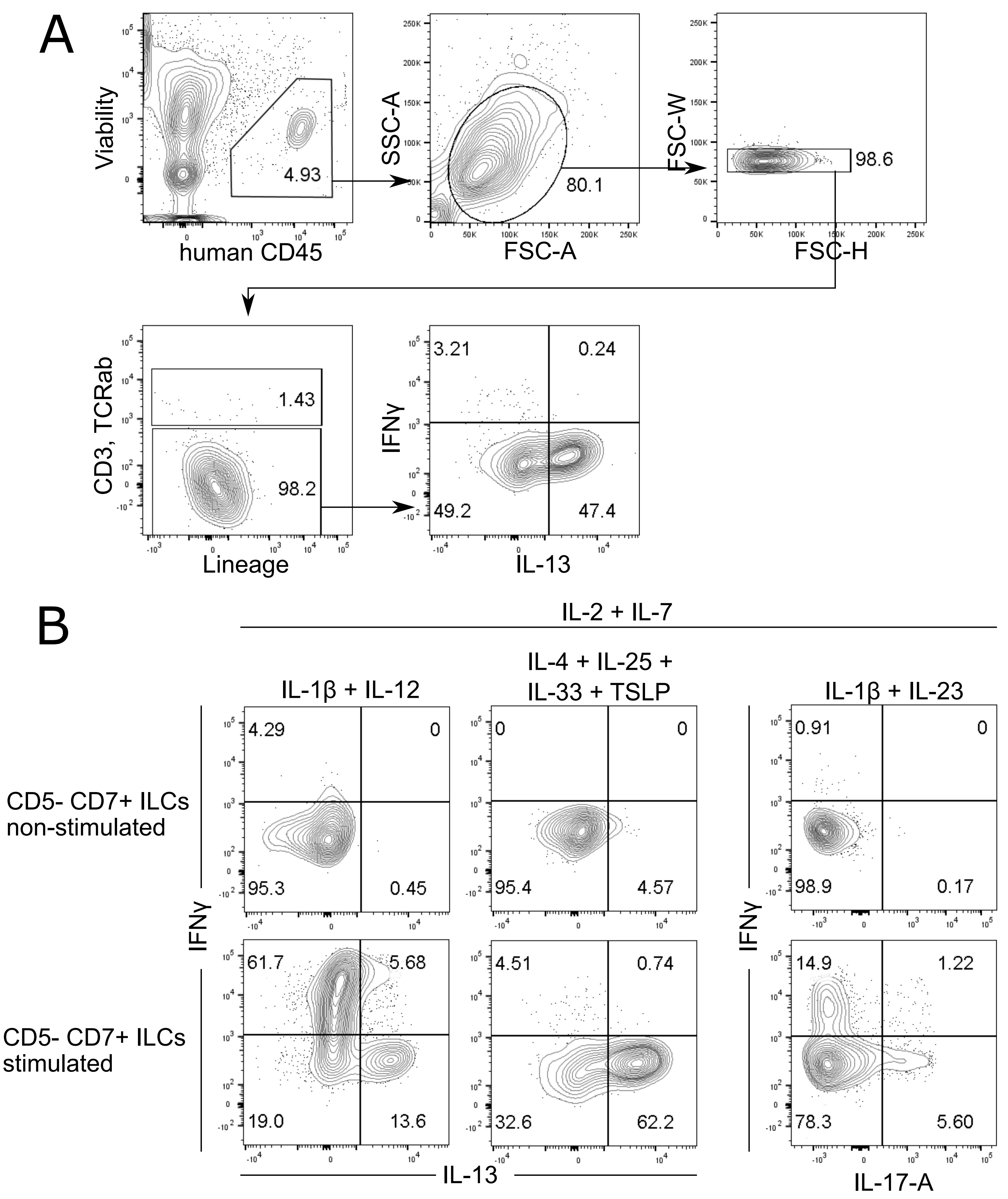

SUPPLEMENTARY FIGURE 3 Gating strategy and non-stimulated controls for intracellular cytokine staining after ILC differentiation in vitro. **(A)** Gating strategy for the flow cytometric analysis of cytokine-producing ILCs one week after co-culture with OP9-DL1 cells as in Figure 4A and B. After co-culture, ILCs were gated as viable $CD45^{+}CD3^{-}TCR\alpha\beta^{-}Lineage^{-}$ cells. **(B)** Non-stimulated controls for the flow cytometry analysis of intracellular cytokine expression by ILCs one week after co-culture with OP9 -DL1 cells in the presence of the indicated polarizing cytokines. Data are representative of at least two experiments.

Figure S4

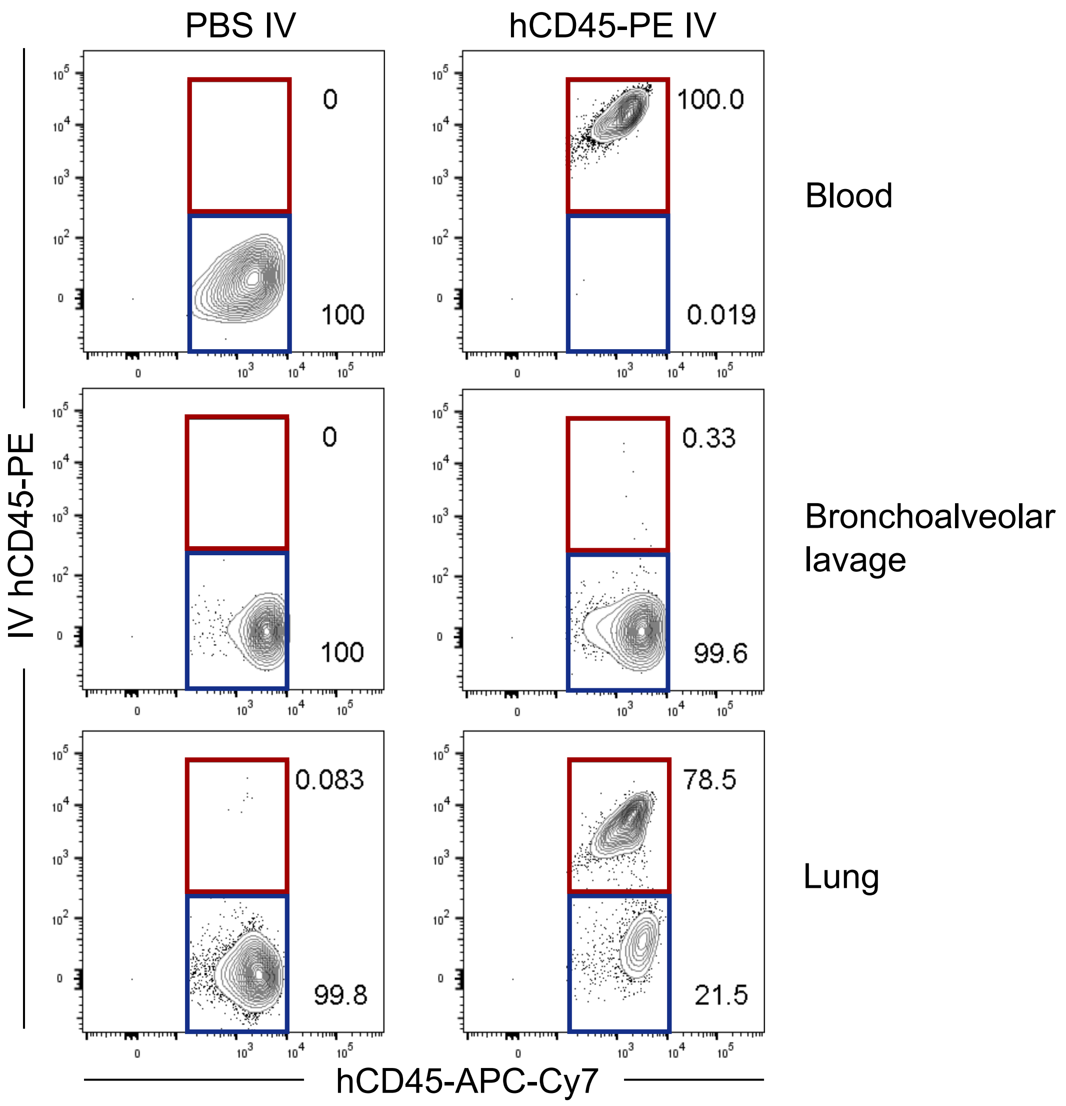

SUPPLEMENTARY FIGURE 4 Specificity of intravascular labeling of human hematopoietic cells in MISTRG mice. Frequencies of intravascular (IV hCD45-PE+) and extravascular (IV hCD45-PE-) human CD45-APC-Cy7+ hematopoietic cells in blood, bronchoalveolar lavage fluid, and lung tissue from MISTRG mice. Adult MISTRG mice engrafted with human CD34+ HSPCs were injected intravenously (IV) with anti-human CD45- PE antibody and tissues harvested 5 minutes later as in Fig. 5A. hCD45, human CD45. Data are representative of two experiments.

Figure S5

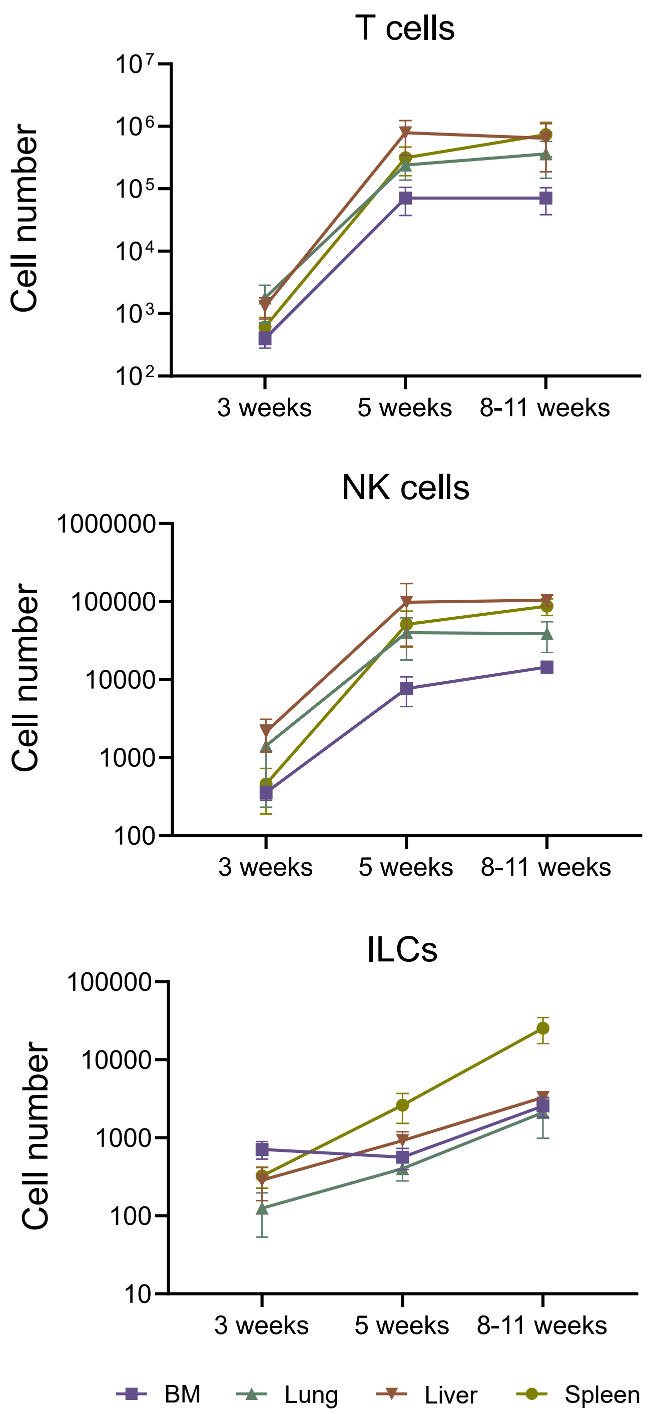

SUPPLEMENTARY FIGURE 5 Kinetics of human T cell, NK cell, and ILC reconstitution in MISTRG mice after transplantation with human CD34$^+$ HSPCs. Numbers of human T cells, NK cells, and ILCs in bone marrow, spleen, liver and lung of MISTRG mice at 3 weeks, 5 weeks and 8-10 weeks post-transplantation with human CD34$^+$ HSPCs (n = 6-9). Data represent mean ± SEM and are representative for at least two experiments.

Figure S6

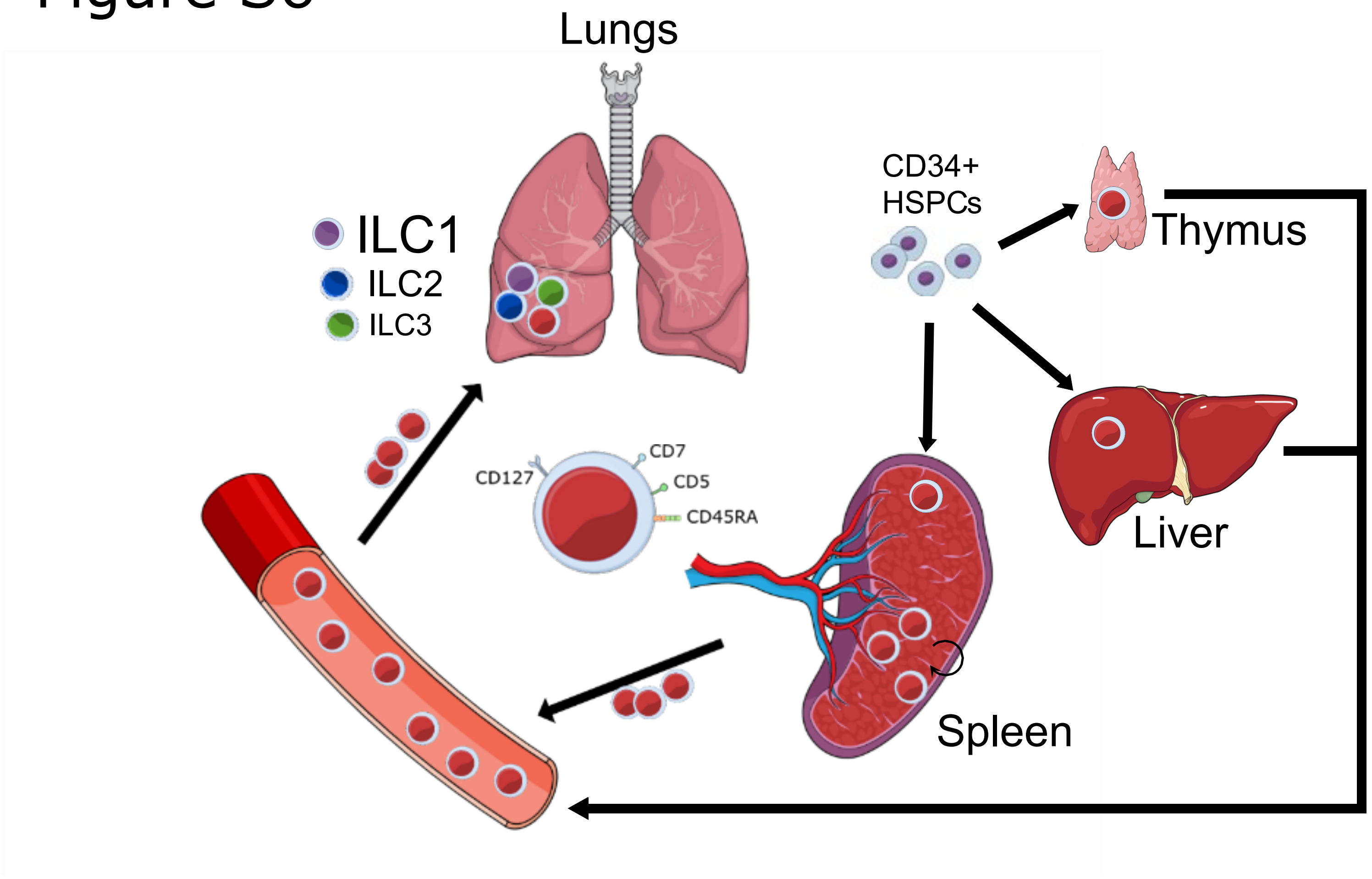

SUPPLEMENTARY FIGURE 6 Model of human CD5$^+$CD7$^+$ ILC development and migration. In humanized MISTRG mice, CD5$^+$CD7$^+$ ILCs develop from human CD34$^+$ HSPCs and take up residence early in the thymus, liver and spleen. Subsequently, CD5$^+$CD7$^+$ ILCs migrate to the lung via the blood. CD5$^+$CD7$^+$ ILCs express the "naïve" surface marker CD45RA and have the ability to produce ILC1, ILC2, and, to a lesser extent, ILC3 cytokines upon stimulation.
